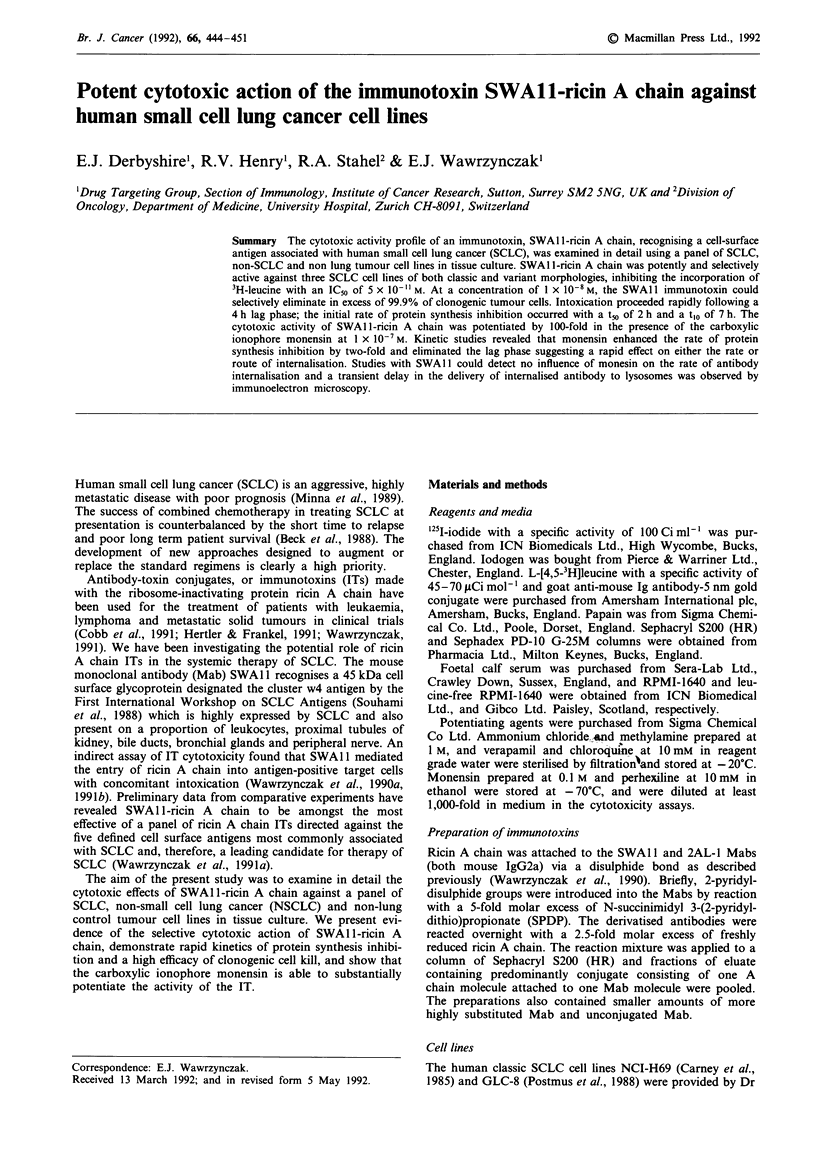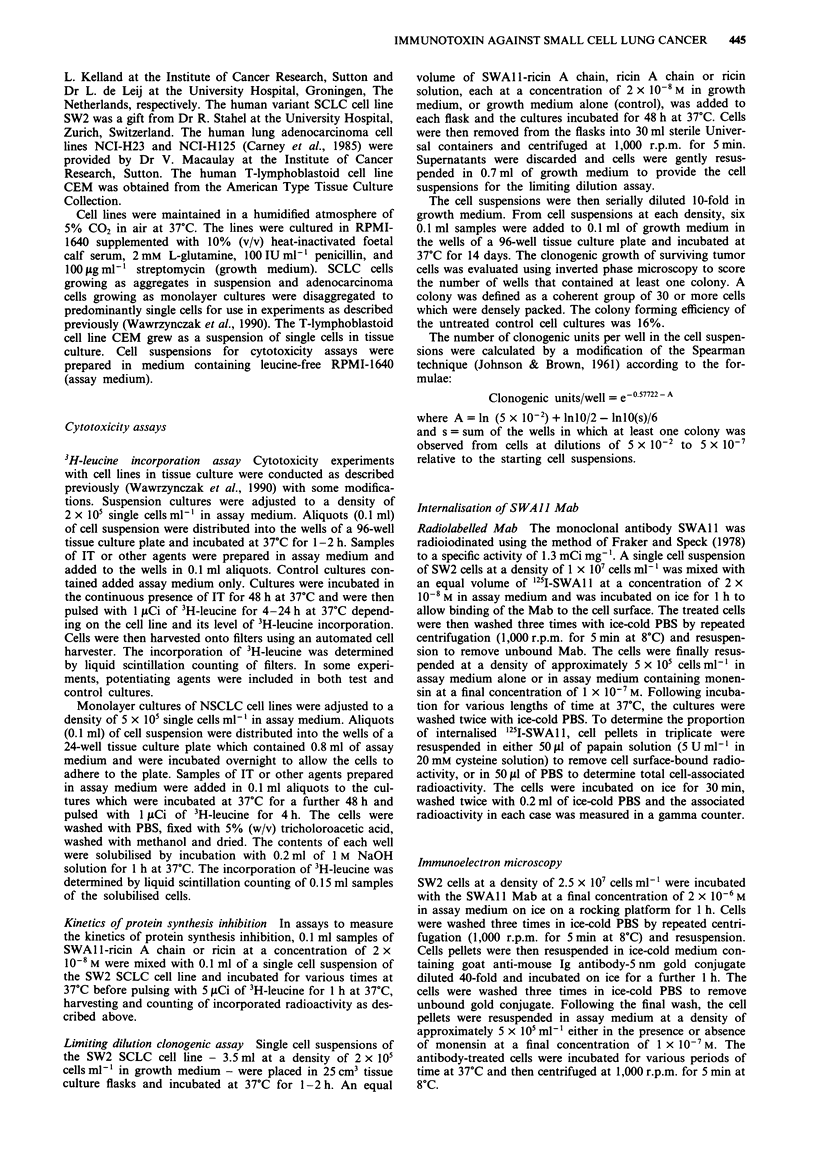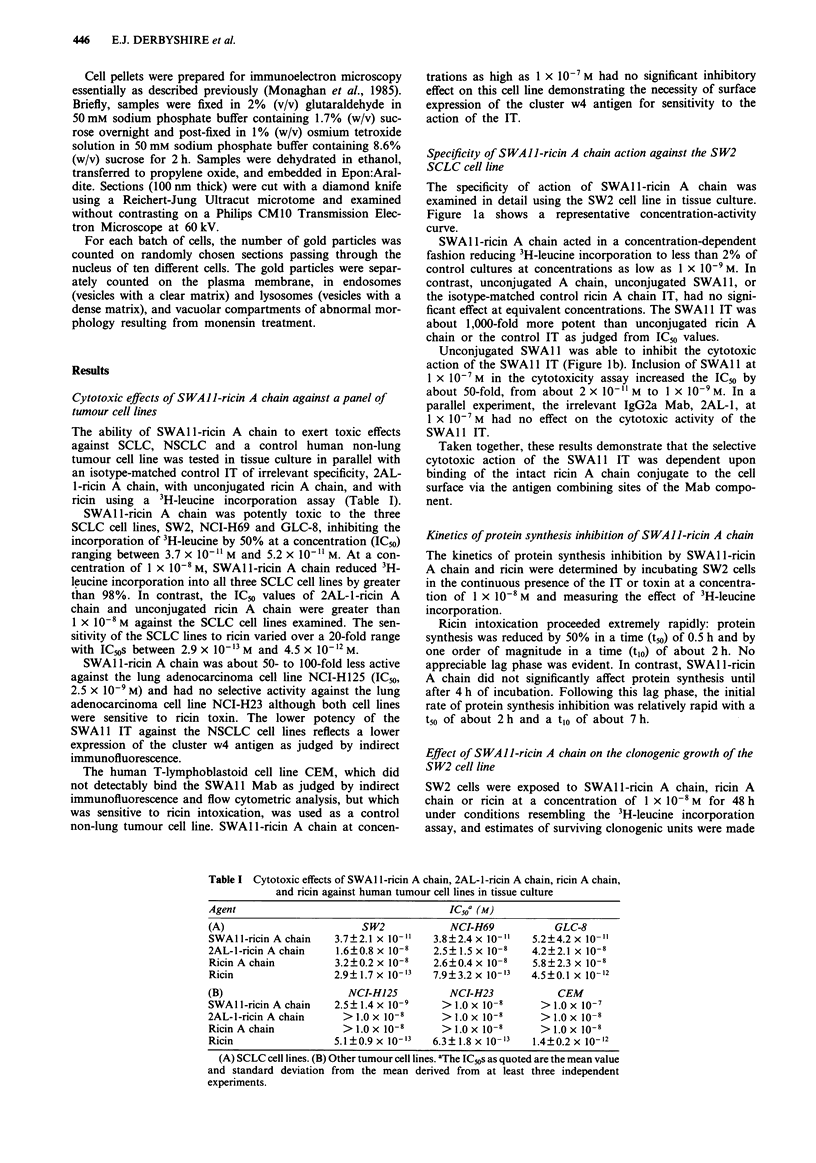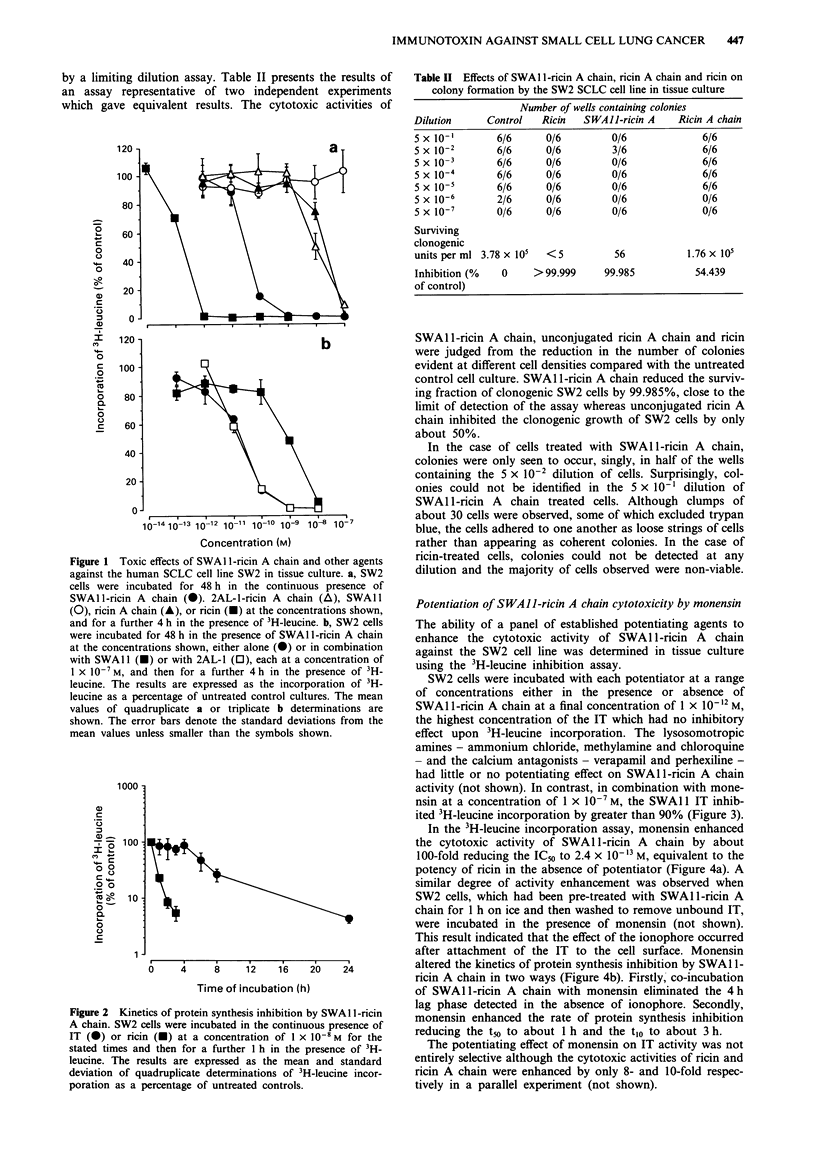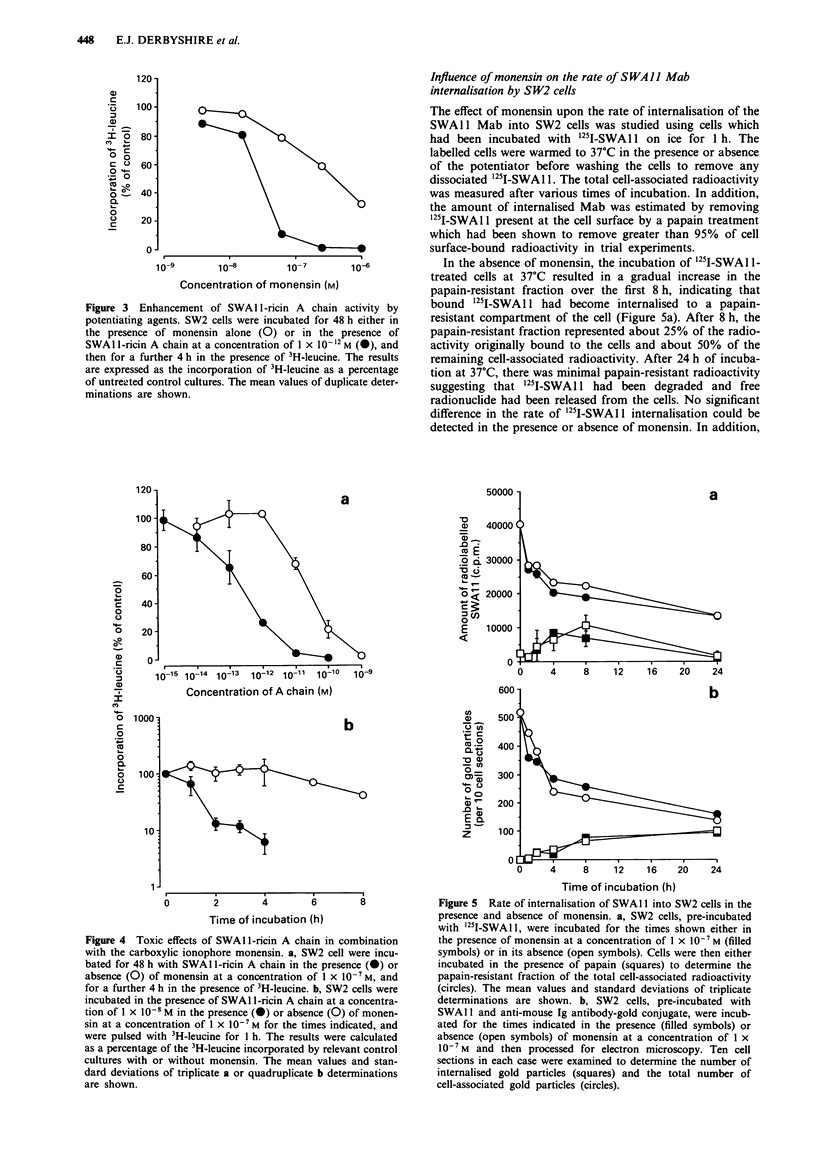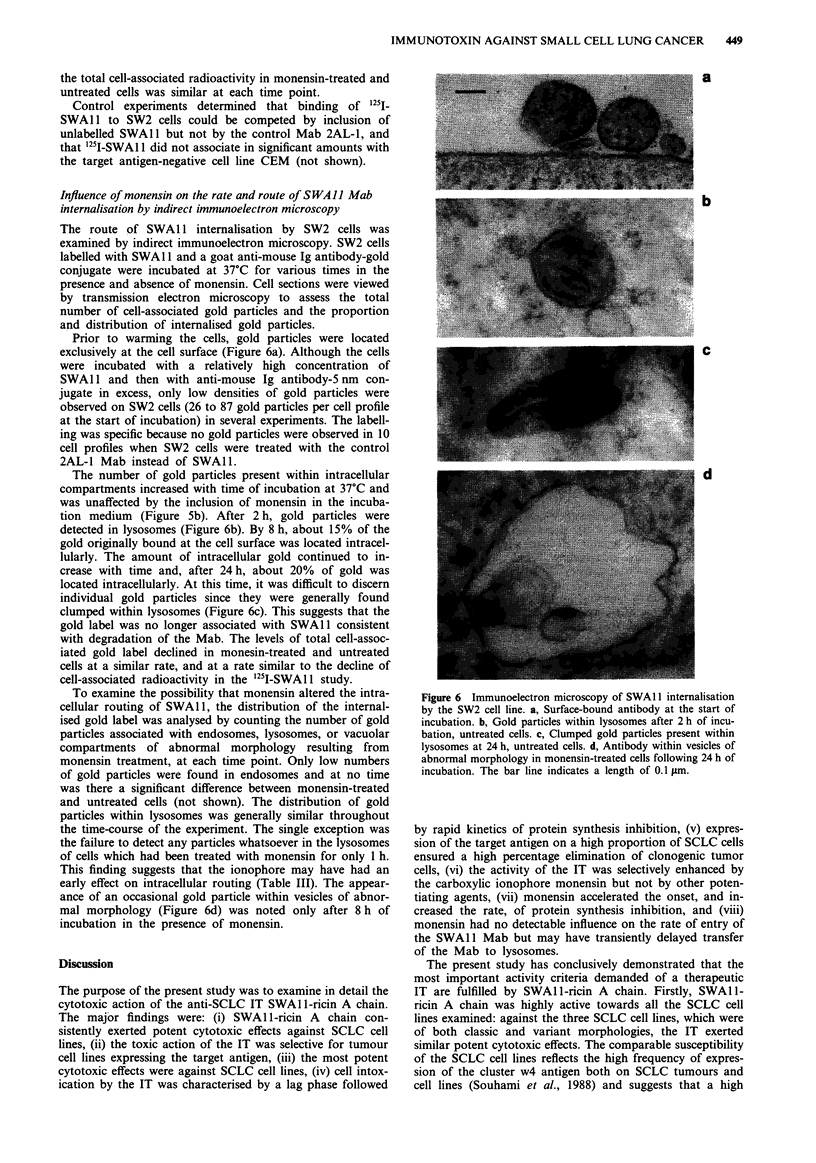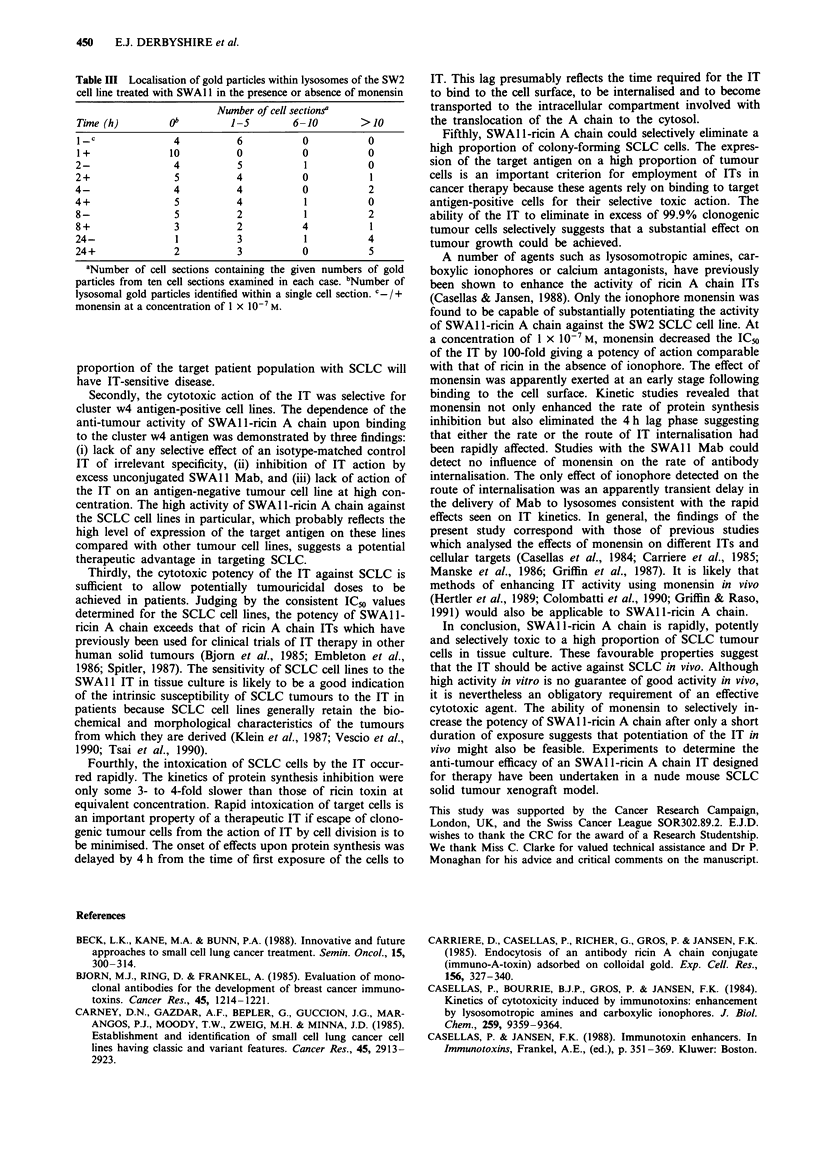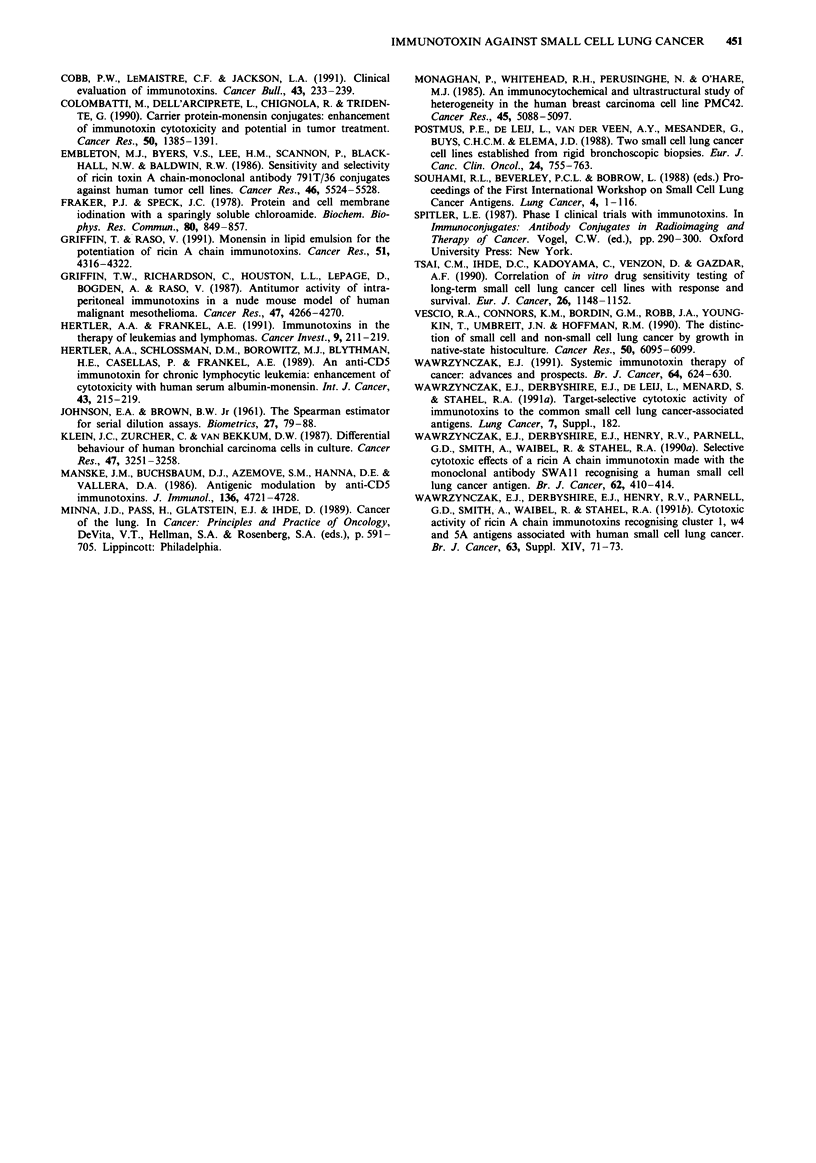# Potent cytotoxic action of the immunotoxin SWA11-ricin A chain against human small cell lung cancer cell lines.

**DOI:** 10.1038/bjc.1992.294

**Published:** 1992-09

**Authors:** E. J. Derbyshire, R. V. Henry, R. A. Stahel, E. J. Wawrzynczak

**Affiliations:** Section of Immunology, Institute of Cancer Research, Sutton, Surrey, UK.

## Abstract

**Images:**


*Br. J. Cancer* (1992), **66**, 444–451 © Macmillan Press Ltd., 1992

### Potent cytotoxic action of the immunotoxin SWA11-ricin A chain against human small cell lung cancer cell lines

E.J. Derbyshire[1], R.V. Henry[1], R.A. Stahel[2] & E.J. Wawrzynczak[1]

[1]*Drug Targeting Group, Section of Immunology, Institute of Cancer Research, Sutton, Surrey SM2 5NG, UK and* [2]*Division of Oncology, Department of Medicine, University Hospital, Zurich CH-8091, Switzerland*

**Summary** The cytotoxic activity profile of an immunotoxin, SWA11-ricin A chain, recognising a cell-surface antigen associated with human small cell lung cancer (SCLC), was examined in detail using a panel of SCLC, non-SCLC and non lung tumour cell lines in tissue culture. SWA11-ricin A chain was potently and selectively active against three SCLC cell lines of both classic and variant morphologies, inhibiting the incorporation of $^{3}$H-leucine with an $IC_{50}$ of $5 \times 10^{-11}$ M. At a concentration of $1 \times 10^{-8}$ M, the SWA11 immunotoxin could selectively eliminate in excess of 99.9% of clonogenic tumour cells. Intoxication proceeded rapidly following a 4 h lag phase; the initial rate of protein synthesis inhibition occurred with a $t_{50}$ of 2 h and a $t_{10}$ of 7 h. The cytotoxic activity of SWA11-ricin A chain was potentiated by 100-fold in the presence of the carboxylic ionophore monensin at $1 \times 10^{-7}$ M. Kinetic studies revealed that monensin enhanced the rate of protein synthesis inhibition by two-fold and eliminated the lag phase suggesting a rapid effect on either the rate or route of internalisation. Studies with SWA11 could detect no influence of monesin on the rate of antibody internalisation and a transient delay in the delivery of internalised antibody to lysosomes was observed by immunoelectron microscopy.

Human small cell lung cancer (SCLC) is an aggressive, highly metastatic disease with poor prognosis (Minna *et al.*, 1989). The success of combined chemotherapy in treating SCLC at presentation is counterbalanced by the short time to relapse and poor long term patient survival (Beck *et al.*, 1988). The development of new approaches designed to augment or replace the standard regimens is clearly a high priority.

Antibody-toxin conjugates, or immunotoxins (ITs) made with the ribosome-inactivating protein ricin A chain have been used for the treatment of patients with leukaemia, lymphoma and metastatic solid tumours in clinical trials (Cobb *et al.*, 1991; Hertler & Frankel, 1991; Wawrzynczak, 1991). We have been investigating the potential role of ricin A chain ITs in the systemic therapy of SCLC. The mouse monoclonal antibody (Mab) SWA11 recognises a 45 kDa cell surface glycoprotein designated the cluster w4 antigen by the First International Workshop on SCLC Antigens (Souhami *et al.*, 1988) which is highly expressed by SCLC and also present on a proportion of leukocytes, proximal tubules of kidney, bile ducts, bronchial glands and peripheral nerve. An indirect assay of IT cytotoxicity found that SWA11 mediated the entry of ricin A chain into antigen-positive target cells with concomitant intoxication (Wawrzynczak *et al.*, 1990*a*, 1991*b*). Preliminary data from comparative experiments have revealed SWA11-ricin A chain to be amongst the most effective of a panel of ricin A chain ITs directed against the five defined cell surface antigens most commonly associated with SCLC and, therefore, a leading candidate for therapy of SCLC (Wawrzynczak *et al.*, 1991*a*).

The aim of the present study was to examine in detail the cytotoxic effects of SWA11-ricin A chain against a panel of SCLC, non-small cell lung cancer (NSCLC) and non-lung control tumour cell lines in tissue culture. We present evidence of the selective cytotoxic action of SWA11-ricin A chain, demonstrate rapid kinetics of protein synthesis inhibition and a high efficacy of clonogenic cell kill, and show that the carboxylic ionophore monensin is able to substantially potentiate the activity of the IT.

Correspondence: E.J. Wawrzynczak.
Received 13 March 1992; and in revised form 5 May 1992.

### Materials and methods

### *Reagents and media*

$^{125}$I-iodide with a specific activity of 100 Ci ml$^{-1}$ was purchased from ICN Biomedicals Ltd., High Wycombe, Bucks, England. Iodogen was bought from Pierce & Warriner Ltd., Chester, England. L-[4,5-$^{3}$H]leucine with a specific activity of 45–70 $\mu$Ci mol$^{-1}$ and goat anti-mouse Ig antibody-5 nm gold conjugate were purchased from Amersham International plc, Amersham, Bucks, England. Papain was from Sigma Chemical Co. Ltd., Poole, Dorset, England. Sephacryl S200 (HR) and Sephadex PD-10 G-25M columns were obtained from Pharmacia Ltd., Milton Keynes, Bucks, England.

Foetal calf serum was purchased from Sera-Lab Ltd., Crawley Down, Sussex, England, and RPMI-1640 and leucine-free RPMI-1640 were obtained from ICN Biomedical Ltd., and Gibco Ltd. Paisley, Scotland, respectively.

Potentiating agents were purchased from Sigma Chemical Co Ltd. Ammonium chloride and methylamine prepared at 1 M, and verapamil and chloroquine at 10 mM in reagent grade water were sterilised by filtration and stored at −20°C. Monensin prepared at 0.1 M and perhexiline at 10 mM in ethanol were stored at −70°C, and were diluted at least 1,000-fold in medium in the cytotoxicity assays.

### *Preparation of immunotoxins*

Ricin A chain was attached to the SWA11 and 2AL-1 Mabs (both mouse IgG2a) via a disulphide bond as described previously (Wawrzynczak *et al.*, 1990). Briefly, 2-pyridyl-disulphide groups were introduced into the Mabs by reaction with a 5-fold molar excess of N-succinimidyl 3-(2-pyridyl-dithio)propionate (SPDP). The derivatised antibodies were reacted overnight with a 2.5-fold molar excess of freshly reduced ricin A chain. The reaction mixture was applied to a column of Sephacryl S200 (HR) and fractions of eluate containing predominantly conjugate consisting of one A chain molecule attached to one Mab molecule were pooled. The preparations also contained smaller amounts of more highly substituted Mab and unconjugated Mab.

### *Cell lines*

The human classic SCLC cell lines NCI-H69 (Carney *et al.*, 1985) and GLC-8 (Postmus *et al.*, 1988) were provided by Dr

L. Kelland at the Institute of Cancer Research, Sutton and Dr L. de Leij at the University Hospital, Groningen, The Netherlands, respectively. The human variant SCLC cell line SW2 was a gift from Dr R. Stahel at the University Hospital, Zurich, Switzerland. The human lung adenocarcinoma cell lines NCI-H23 and NCI-H125 (Carney *et al.*, 1985) were provided by Dr V. Macaulay at the Institute of Cancer Research, Sutton. The human T-lymphoblastoid cell line CEM was obtained from the American Type Tissue Culture Collection.

Cell lines were maintained in a humidified atmosphere of 5% $CO_2$ in air at 37°C. The lines were cultured in RPMI-1640 supplemented with 10% (v/v) heat-inactivated foetal calf serum, 2 mM L-glutamine, 100 IU $ml^{-1}$ penicillin, and 100 $\mu g\ ml^{-1}$ streptomycin (growth medium). SCLC cells growing as aggregates in suspension and adenocarcinoma cells growing as monolayer cultures were disaggregated to predominantly single cells for use in experiments as described previously (Wawrzynczak *et al.*, 1990). The T-lymphoblastoid cell line CEM grew as a suspension of single cells in tissue culture. Cell suspensions for cytotoxicity assays were prepared in medium containing leucine-free RPMI-1640 (assay medium).

### *Cytotoxicity assays*

*$^3$H-leucine incorporation assay* Cytotoxicity experiments with cell lines in tissue culture were conducted as described previously (Wawrzynczak *et al.*, 1990) with some modifications. Suspension cultures were adjusted to a density of $2 \times 10^5$ single cells $ml^{-1}$ in assay medium. Aliquots (0.1 ml) of cell suspension were distributed into the wells of a 96-well tissue culture plate and incubated at 37°C for 1–2 h. Samples of IT or other agents were prepared in assay medium and added to the wells in 0.1 ml aliquots. Control cultures contained added assay medium only. Cultures were incubated in the continuous presence of IT for 48 h at 37°C and were then pulsed with 1 $\mu$Ci of $^3$H-leucine for 4–24 h at 37°C depending on the cell line and its level of $^3$H-leucine incorporation. Cells were then harvested onto filters using an automated cell harvester. The incorporation of $^3$H-leucine was determined by liquid scintillation counting of filters. In some experiments, potentiating agents were included in both test and control cultures.

Monolayer cultures of NSCLC cell lines were adjusted to a density of $5 \times 10^5$ single cells $ml^{-1}$ in assay medium. Aliquots (0.1 ml) of cell suspension were distributed into the wells of a 24-well tissue culture plate which contained 0.8 ml of assay medium and were incubated overnight to allow the cells to adhere to the plate. Samples of IT or other agents prepared in assay medium were added in 0.1 ml aliquots to the cultures which were incubated at 37°C for a further 48 h and pulsed with 1 $\mu$Ci of $^3$H-leucine for 4 h. The cells were washed with PBS, fixed with 5% (w/v) tricholoroacetic acid, washed with methanol and dried. The contents of each well were solubilised by incubation with 0.2 ml of 1 M NaOH solution for 1 h at 37°C. The incorporation of $^3$H-leucine was determined by liquid scintillation counting of 0.15 ml samples of the solubilised cells.

*Kinetics of protein synthesis inhibition* In assays to measure the kinetics of protein synthesis inhibition, 0.1 ml samples of SWA11-ricin A chain or ricin at a concentration of $2 \times 10^{-8}$ M were mixed with 0.1 ml of a single cell suspension of the SW2 SCLC cell line and incubated for various times at 37°C before pulsing with 5 $\mu$Ci of $^3$H-leucine for 1 h at 37°C, harvesting and counting of incorporated radioactivity as described above.

*Limiting dilution clonogenic assay* Single cell suspensions of the SW2 SCLC cell line – 3.5 ml at a density of $2 \times 10^5$ cells $ml^{-1}$ in growth medium – were placed in 25 $cm^3$ tissue culture flasks and incubated at 37°C for 1–2 h. An equal volume of SWA11-ricin A chain, ricin A chain or ricin solution, each at a concentration of $2 \times 10^{-8}$ M in growth medium, or growth medium alone (control), was added to each flask and the cultures incubated for 48 h at 37°C. Cells were then removed from the flasks into 30 ml sterile Universal containers and centrifuged at 1,000 r.p.m. for 5 min. Supernatants were discarded and cells were gently resuspended in 0.7 ml of growth medium to provide the cell suspensions for the limiting dilution assay.

The cell suspensions were then serially diluted 10-fold in growth medium. From cell suspensions at each density, six 0.1 ml samples were added to 0.1 ml of growth medium in the wells of a 96-well tissue culture plate and incubated at 37°C for 14 days. The clonogenic growth of surviving tumor cells was evaluated using inverted phase microscopy to score the number of wells that contained at least one colony. A colony was defined as a coherent group of 30 or more cells which were densely packed. The colony forming efficiency of the untreated control cell cultures was 16%.

The number of clonogenic units per well in the cell suspensions were calculated by a modification of the Spearman technique (Johnson & Brown, 1961) according to the formulae:

$$\text{Clonogenic units/well} = e^{-0.57722 - A}$$

where $A = \ln\ (5 \times 10^{-2}) + \ln 10/2 - \ln 10(s)/6$

and s = sum of the wells in which at least one colony was observed from cells at dilutions of $5 \times 10^{-2}$ to $5 \times 10^{-7}$ relative to the starting cell suspensions.

### *Internalisation of SWA11 Mab*

*Radiolabelled Mab* The monoclonal antibody SWA11 was radioiodinated using the method of Fraker and Speck (1978) to a specific activity of 1.3 mCi $mg^{-1}$. A single cell suspension of SW2 cells at a density of $1 \times 10^7$ cells $ml^{-1}$ was mixed with an equal volume of $^{125}$I-SWA11 at a concentration of $2 \times 10^{-8}$ M in assay medium and was incubated on ice for 1 h to allow binding of the Mab to the cell surface. The treated cells were then washed three times with ice-cold PBS by repeated centrifugation (1,000 r.p.m. for 5 min at 8°C) and resuspension to remove unbound Mab. The cells were finally resuspended at a density of approximately $5 \times 10^5$ cells $ml^{-1}$ in assay medium alone or in assay medium containing monensin at a final concentration of $1 \times 10^{-7}$ M. Following incubation for various lengths of time at 37°C, the cultures were washed twice with ice-cold PBS. To determine the proportion of internalised $^{125}$I-SWA11, cell pellets in triplicate were resuspended in either 50 $\mu$l of papain solution (5 U $ml^{-1}$ in 20 mM cysteine solution) to remove cell surface-bound radioactivity, or in 50 $\mu$l of PBS to determine total cell-associated radioactivity. The cells were incubated on ice for 30 min, washed twice with 0.2 ml of ice-cold PBS and the associated radioactivity in each case was measured in a gamma counter.

### *Immunoelectron microscopy*

SW2 cells at a density of $2.5 \times 10^7$ cells $ml^{-1}$ were incubated with the SWA11 Mab at a final concentration of $2 \times 10^{-6}$ M in assay medium on ice on a rocking platform for 1 h. Cells were washed three times in ice-cold PBS by repeated centrifugation (1,000 r.p.m. for 5 min at 8°C) and resuspension. Cells pellets were then resuspended in ice-cold medium containing goat anti-mouse Ig antibody-5 nm gold conjugate diluted 40-fold and incubated on ice for a further 1 h. The cells were washed three times in ice-cold PBS to remove unbound gold conjugate. Following the final wash, the cell pellets were resuspended in assay medium at a density of approximately $5 \times 10^5$ $ml^{-1}$ either in the presence or absence of monensin at a final concentration of $1 \times 10^{-7}$ M. The antibody-treated cells were incubated for various periods of time at 37°C and then centrifuged at 1,000 r.p.m. for 5 min at 8°C.

Cell pellets were prepared for immunoelectron microscopy essentially as described previously (Monaghan *et al.*, 1985). Briefly, samples were fixed in 2% (v/v) glutaraldehyde in 50 mM sodium phosphate buffer containing 1.7% (w/v) sucrose overnight and post-fixed in 1% (w/v) osmium tetroxide solution in 50 mM sodium phosphate buffer containing 8.6% (w/v) sucrose for 2 h. Samples were dehydrated in ethanol, transferred to propylene oxide, and embedded in Epon:Araldite. Sections (100 nm thick) were cut with a diamond knife using a Reichert-Jung Ultracut microtome and examined without contrasting on a Philips CM10 Transmission Electron Microscope at 60 kV.

For each batch of cells, the number of gold particles was counted on randomly chosen sections passing through the nucleus of ten different cells. The gold particles were separately counted on the plasma membrane, in endosomes (vesicles with a clear matrix) and lysosomes (vesicles with a dense matrix), and vacuolar compartments of abnormal morphology resulting from monensin treatment.

### Results

### *Cytotoxic effects of SWA11-ricin A chain against a panel of tumour cell lines*

The ability of SWA11-ricin A chain to exert toxic effects against SCLC, NSCLC and a control human non-lung tumour cell line was tested in tissue culture in parallel with an isotype-matched control IT of irrelevant specificity, 2AL-1-ricin A chain, with unconjugated ricin A chain, and with ricin using a $^3$H-leucine incorporation assay (Table I).

SWA11-ricin A chain was potently toxic to the three SCLC cell lines, SW2, NCI-H69 and GLC-8, inhibiting the incorporation of $^3$H-leucine by 50% at a concentration ($IC_{50}$) ranging between $3.7 \times 10^{-11}$ M and $5.2 \times 10^{-11}$ M. At a concentration of $1 \times 10^{-8}$ M, SWA11-ricin A chain reduced $^3$H-leucine incorporation into all three SCLC cell lines by greater than 98%. In contrast, the $IC_{50}$ values of 2AL-1-ricin A chain and unconjugated ricin A chain were greater than $1 \times 10^{-8}$ M against the SCLC cell lines examined. The sensitivity of the SCLC lines to ricin varied over a 20-fold range with $IC_{50}$s between $2.9 \times 10^{-13}$ M and $4.5 \times 10^{-12}$ M.

SWA11-ricin A chain was about 50- to 100-fold less active against the lung adenocarcinoma cell line NCI-H125 ($IC_{50}$, $2.5 \times 10^{-9}$ M) and had no selective activity against the lung adenocarcinoma cell line NCI-H23 although both cell lines were sensitive to ricin toxin. The lower potency of the SWA11 IT against the NSCLC cell lines reflects a lower expression of the cluster w4 antigen as judged by indirect immunofluorescence.

The human T-lymphoblastoid cell line CEM, which did not detectably bind the SWA11 Mab as judged by indirect immunofluorescence and flow cytometric analysis, but which was sensitive to ricin intoxication, was used as a control non-lung tumour cell line. SWA11-ricin A chain at concentrations as high as $1 \times 10^{-7}$ M had no significant inhibitory effect on this cell line demonstrating the necessity of surface expression of the cluster w4 antigen for sensitivity to the action of the IT.

### *Specificity of SWA11-ricin A chain action against the SW2 SCLC cell line*

The specificity of action of SWA11-ricin A chain was examined in detail using the SW2 cell line in tissue culture. Figure 1a shows a representative concentration-activity curve.

SWA11-ricin A chain acted in a concentration-dependent fashion reducing $^3$H-leucine incorporation to less than 2% of control cultures at concentrations as low as $1 \times 10^{-9}$ M. In contrast, unconjugated A chain, unconjugated SWA11, or the isotype-matched control ricin A chain IT, had no significant effect at equivalent concentrations. The SWA11 IT was about 1,000-fold more potent than unconjugated ricin A chain or the control IT as judged from $IC_{50}$ values.

Unconjugated SWA11 was able to inhibit the cytotoxic action of the SWA11 IT (Figure 1b). Inclusion of SWA11 at $1 \times 10^{-7}$ M in the cytotoxicity assay increased the $IC_{50}$ by about 50-fold, from about $2 \times 10^{-11}$ M to $1 \times 10^{-9}$ M. In a parallel experiment, the irrelevant IgG2a Mab, 2AL-1, at $1 \times 10^{-7}$ M had no effect on the cytotoxic activity of the SWA11 IT.

Taken together, these results demonstrate that the selective cytotoxic action of the SWA11 IT was dependent upon binding of the intact ricin A chain conjugate to the cell surface via the antigen combining sites of the Mab component.

### *Kinetics of protein synthesis inhibition of SWA11-ricin A chain*

The kinetics of protein synthesis inhibition by SWA11-ricin A chain and ricin were determined by incubating SW2 cells in the continuous presence of the IT or toxin at a concentration of $1 \times 10^{-8}$ M and measuring the effect of $^3$H-leucine incorporation.

Ricin intoxication proceeded extremely rapidly: protein synthesis was reduced by 50% in a time ($t_{50}$) of 0.5 h and by one order of magnitude in a time ($t_{10}$) of about 2 h. No appreciable lag phase was evident. In contrast, SWA11-ricin A chain did not significantly affect protein synthesis until after 4 h of incubation. Following this lag phase, the initial rate of protein synthesis inhibition was relatively rapid with a $t_{50}$ of about 2 h and a $t_{10}$ of about 7 h.

### *Effect of SWA11-ricin A chain on the clonogenic growth of the SW2 cell line*

SW2 cells were exposed to SWA11-ricin A chain, ricin A chain or ricin at a concentration of $1 \times 10^{-8}$ M for 48 h under conditions resembling the $^3$H-leucine incorporation assay, and estimates of surviving clonogenic units were made

**Table I** Cytotoxic effects of SWA11-ricin A chain, 2AL-1-ricin A chain, ricin A chain, and ricin against human tumour cell lines in tissue culture

| *Agent* | $IC_{50}$[a] (M) | | |
|---|---|---|---|
| (A) | *SW2* | *NCI-H69* | *GLC-8* |
| SWA11-ricin A chain | $3.7 \pm 2.1 \times 10^{-11}$ | $3.8 \pm 2.4 \times 10^{-11}$ | $5.2 \pm 4.2 \times 10^{-11}$ |
| 2AL-1-ricin A chain | $1.6 \pm 0.8 \times 10^{-8}$ | $2.5 \pm 1.5 \times 10^{-8}$ | $4.2 \pm 2.1 \times 10^{-8}$ |
| Ricin A chain | $3.2 \pm 0.2 \times 10^{-8}$ | $2.6 \pm 0.4 \times 10^{-8}$ | $5.8 \pm 2.3 \times 10^{-8}$ |
| Ricin | $2.9 \pm 1.7 \times 10^{-13}$ | $7.9 \pm 3.2 \times 10^{-13}$ | $4.5 \pm 0.1 \times 10^{-12}$ |
| (B) | *NCI-H125* | *NCI-H23* | *CEM* |
| SWA11-ricin A chain | $2.5 \pm 1.4 \times 10^{-9}$ | $> 1.0 \times 10^{-8}$ | $> 1.0 \times 10^{-7}$ |
| 2AL-1-ricin A chain | $> 1.0 \times 10^{-8}$ | $> 1.0 \times 10^{-8}$ | $> 1.0 \times 10^{-8}$ |
| Ricin A chain | $> 1.0 \times 10^{-8}$ | $> 1.0 \times 10^{-8}$ | $> 1.0 \times 10^{-8}$ |
| Ricin | $5.1 \pm 0.9 \times 10^{-13}$ | $6.3 \pm 1.8 \times 10^{-13}$ | $1.4 \pm 0.2 \times 10^{-12}$ |

(A) SCLC cell lines. (B) Other tumour cell lines. [a]The $IC_{50}$s as quoted are the mean value and standard deviation from the mean derived from at least three independent experiments.

by a limiting dilution assay. Table II presents the results of an assay representative of two independent experiments which gave equivalent results. The cytotoxic activities of

**Figure 1** Toxic effects of SWA11-ricin A chain and other agents against the human SCLC cell line SW2 in tissue culture. **a**, SW2 cells were incubated for 48 h in the continuous presence of SWA11-ricin A chain (●). 2AL-1-ricin A chain (△), SWA11 (○), ricin A chain (▲), or ricin (■) at the concentrations shown, and for a further 4 h in the presence of $^3$H-leucine. **b**, SW2 cells were incubated for 48 h in the presence of SWA11-ricin A chain at the concentrations shown, either alone (●) or in combination with SWA11 (■) or with 2AL-1 (□), each at a concentration of $1 \times 10^{-7}$ M, and then for a further 4 h in the presence of $^3$H-leucine. The results are expressed as the incorporation of $^3$H-leucine as a percentage of untreated control cultures. The mean values of quadruplicate **a** or triplicate **b** determinations are shown. The error bars denote the standard deviations from the mean values unless smaller than the symbols shown.

**Figure 2** Kinetics of protein synthesis inhibition by SWA11-ricin A chain. SW2 cells were incubated in the continuous presence of IT (●) or ricin (■) at a concentration of $1 \times 10^{-8}$ M for the stated times and then for a further 1 h in the presence of $^3$H-leucine. The results are expressed as the mean and standard deviation of quadruplicate determinations of $^3$H-leucine incorporation as a percentage of untreated controls.

**Table II** Effects of SWA11-ricin A chain, ricin A chain and ricin on colony formation by the SW2 SCLC cell line in tissue culture

| | *Number of wells containing colonies* | | | |
|---|---|---|---|---|
| *Dilution* | *Control* | *Ricin* | *SWA11-ricin A* | *Ricin A chain* |
| $5 \times 10^{-1}$ | 6/6 | 0/6 | 0/6 | 6/6 |
| $5 \times 10^{-2}$ | 6/6 | 0/6 | 3/6 | 6/6 |
| $5 \times 10^{-3}$ | 6/6 | 0/6 | 0/6 | 6/6 |
| $5 \times 10^{-4}$ | 6/6 | 0/6 | 0/6 | 6/6 |
| $5 \times 10^{-5}$ | 6/6 | 0/6 | 0/6 | 6/6 |
| $5 \times 10^{-6}$ | 2/6 | 0/6 | 0/6 | 0/6 |
| $5 \times 10^{-7}$ | 0/6 | 0/6 | 0/6 | 0/6 |
| Surviving clonogenic units per ml | $3.78 \times 10^5$ | $<5$ | 56 | $1.76 \times 10^5$ |
| Inhibition (% of control) | 0 | $>99.999$ | 99.985 | 54.439 |

SWA11-ricin A chain, unconjugated ricin A chain and ricin were judged from the reduction in the number of colonies evident at different cell densities compared with the untreated control cell culture. SWA11-ricin A chain reduced the surviving fraction of clonogenic SW2 cells by 99.985%, close to the limit of detection of the assay whereas unconjugated ricin A chain inhibited the clonogenic growth of SW2 cells by only about 50%.

In the case of cells treated with SWA11-ricin A chain, colonies were only seen to occur, singly, in half of the wells containing the $5 \times 10^{-2}$ dilution of cells. Surprisingly, colonies could not be identified in the $5 \times 10^{-1}$ dilution of SWA11-ricin A chain treated cells. Although clumps of about 30 cells were observed, some of which excluded trypan blue, the cells adhered to one another as loose strings of cells rather than appearing as coherent colonies. In the case of ricin-treated cells, colonies could not be detected at any dilution and the majority of cells observed were non-viable.

### *Potentiation of SWA11-ricin A chain cytotoxicity by monensin*

The ability of a panel of established potentiating agents to enhance the cytotoxic activity of SWA11-ricin A chain against the SW2 cell line was determined in tissue culture using the $^3$H-leucine inhibition assay.

SW2 cells were incubated with each potentiator at a range of concentrations either in the presence or absence of SWA11-ricin A chain at a final concentration of $1 \times 10^{-12}$ M, the highest concentration of the IT which had no inhibitory effect upon $^3$H-leucine incorporation. The lysosomotropic amines – ammonium chloride, methylamine and chloroquine – and the calcium antagonists – verapamil and perhexiline – had little or no potentiating effect on SWA11-ricin A chain activity (not shown). In contrast, in combination with monensin at a concentration of $1 \times 10^{-7}$ M, the SWA11 IT inhibited $^3$H-leucine incorporation by greater than 90% (Figure 3).

In the $^3$H-leucine incorporation assay, monensin enhanced the cytotoxic activity of SWA11-ricin A chain by about 100-fold reducing the $IC_{50}$ to $2.4 \times 10^{-13}$ M, equivalent to the potency of ricin in the absence of potentiator (Figure 4a). A similar degree of activity enhancement was observed when SW2 cells, which had been pre-treated with SWA11-ricin A chain for 1 h on ice and then washed to remove unbound IT, were incubated in the presence of monensin (not shown). This result indicated that the effect of the ionophore occurred after attachment of the IT to the cell surface. Monensin altered the kinetics of protein synthesis inhibition by SWA11-ricin A chain in two ways (Figure 4b). Firstly, co-incubation of SWA11-ricin A chain with monensin eliminated the 4 h lag phase detected in the absence of ionophore. Secondly, monensin enhanced the rate of protein synthesis inhibition reducing the $t_{50}$ to about 1 h and the $t_{10}$ to about 3 h.

The potentiating effect of monensin on IT activity was not entirely selective although the cytotoxic activities of ricin and ricin A chain were enhanced by only 8- and 10-fold respectively in a parallel experiment (not shown).

**Figure 3** Enhancement of SWA11-ricin A chain activity by potentiating agents. SW2 cells were incubated for 48 h either in the presence of monensin alone (O) or in the presence of SWA11-ricin A chain at a concentration of $1 \times 10^{-12}$ M (●), and then for a further 4 h in the presence of $^3$H-leucine. The results are expressed as the incorporation of $^3$H-leucine as a percentage of untreated control cultures. The mean values of duplicate determinations are shown.

*Influence of monensin on the rate of SWA11 Mab internalisation by SW2 cells*

The effect of monensin upon the rate of internalisation of the SWA11 Mab into SW2 cells was studied using cells which had been incubated with $^{125}$I-SWA11 on ice for 1 h. The labelled cells were warmed to 37°C in the presence or absence of the potentiator before washing the cells to remove any dissociated $^{125}$I-SWA11. The total cell-associated radioactivity was measured after various times of incubation. In addition, the amount of internalised Mab was estimated by removing $^{125}$I-SWA11 present at the cell surface by a papain treatment which had been shown to remove greater than 95% of cell surface-bound radioactivity in trial experiments.

In the absence of monensin, the incubation of $^{125}$I-SWA11-treated cells at 37°C resulted in a gradual increase in the papain-resistant fraction over the first 8 h, indicating that bound $^{125}$I-SWA11 had become internalised to a papain-resistant compartment of the cell (Figure 5a). After 8 h, the papain-resistant fraction represented about 25% of the radioactivity originally bound to the cells and about 50% of the remaining cell-associated radioactivity. After 24 h of incubation at 37°C, there was minimal papain-resistant radioactivity suggesting that $^{125}$I-SWA11 had been degraded and free radionuclide had been released from the cells. No significant difference in the rate of $^{125}$I-SWA11 internalisation could be detected in the presence or absence of monensin. In addition,

**Figure 4** Toxic effects of SWA11-ricin A chain in combination with the carboxylic ionophore monensin. **a**, SW2 cell were incubated for 48 h with SWA11-ricin A chain in the presence (●) or absence (O) of monensin at a concentration of $1 \times 10^{-7}$ M, and for a further 4 h in the presence of $^3$H-leucine. **b**, SW2 cells were incubated in the presence of SWA11-ricin A chain at a concentration of $1 \times 10^{-8}$ M in the presence (●) or absence (O) of monensin at a concentration of $1 \times 10^{-7}$ M for the times indicated, and were pulsed with $^3$H-leucine for 1 h. The results were calculated as a percentage of the $^3$H-leucine incorporated by relevant control cultures with or without monensin. The mean values and standard deviations of triplicate **a** or quadruplicate **b** determinations are shown.

**Figure 5** Rate of internalisation of SWA11 into SW2 cells in the presence and absence of monensin. **a**, SW2 cells, pre-incubated with $^{125}$I-SWA11, were incubated for the times shown either in the presence of monensin at a concentration of $1 \times 10^{-7}$ M (filled symbols) or in its absence (open symbols). Cells were then either incubated in the presence of papain (squares) to determine the papain-resistant fraction of the total cell-associated radioactivity (circles). The mean values and standard deviations of triplicate determinations are shown. **b**, SW2 cells, pre-incubated with SWA11 and anti-mouse Ig antibody-gold conjugate, were incubated for the times indicated in the presence (filled symbols) or absence (open symbols) of monensin at a concentration of $1 \times 10^{-7}$ M and then processed for electron microscopy. Ten cell sections in each case were examined to determine the number of internalised gold particles (squares) and the total number of cell-associated gold particles (circles).

the total cell-associated radioactivity in monensin-treated and untreated cells was similar at each time point.

Control experiments determined that binding of $^{125}$I-SWA11 to SW2 cells could be competed by inclusion of unlabelled SWA11 but not by the control Mab 2AL-1, and that $^{125}$I-SWA11 did not associate in significant amounts with the target antigen-negative cell line CEM (not shown).

*Influence of monensin on the rate and route of SWA11 Mab internalisation by indirect immunoelectron microscopy*

The route of SWA11 internalisation by SW2 cells was examined by indirect immunoelectron microscopy. SW2 cells labelled with SWA11 and a goat anti-mouse Ig antibody-gold conjugate were incubated at 37°C for various times in the presence and absence of monensin. Cell sections were viewed by transmission electron microscopy to assess the total number of cell-associated gold particles and the proportion and distribution of internalised gold particles.

Prior to warming the cells, gold particles were located exclusively at the cell surface (Figure 6a). Although the cells were incubated with a relatively high concentration of SWA11 and then with anti-mouse Ig antibody-5 nm conjugate in excess, only low densities of gold particles were observed on SW2 cells (26 to 87 gold particles per cell profile at the start of incubation) in several experiments. The labelling was specific because no gold particles were observed in 10 cell profiles when SW2 cells were treated with the control 2AL-1 Mab instead of SWA11.

The number of gold particles present within intracellular compartments increased with time of incubation at 37°C and was unaffected by the inclusion of monensin in the incubation medium (Figure 5b). After 2 h, gold particles were detected in lysosomes (Figure 6b). By 8 h, about 15% of the gold originally bound at the cell surface was located intracellularly. The amount of intracellular gold continued to increase with time and, after 24 h, about 20% of gold was located intracellularly. At this time, it was difficult to discern individual gold particles since they were generally found clumped within lysosomes (Figure 6c). This suggests that the gold label was no longer associated with SWA11 consistent with degradation of the Mab. The levels of total cell-associated gold label declined in monesin-treated and untreated cells at a similar rate, and at a rate similar to the decline of cell-associated radioactivity in the $^{125}$I-SWA11 study.

To examine the possibility that monensin altered the intracellular routing of SWA11, the distribution of the internalised gold label was analysed by counting the number of gold particles associated with endosomes, lysosomes, or vacuolar compartments of abnormal morphology resulting from monensin treatment, at each time point. Only low numbers of gold particles were found in endosomes and at no time was there a significant difference between monensin-treated and untreated cells (not shown). The distribution of gold particles within lysosomes was generally similar throughout the time-course of the experiment. The single exception was the failure to detect any particles whatsoever in the lysosomes of cells which had been treated with monensin for only 1 h. This finding suggests that the ionophore may have had an early effect on intracellular routing (Table III). The appearance of an occasional gold particle within vesicles of abnormal morphology (Figure 6d) was noted only after 8 h of incubation in the presence of monensin.

**Figure 6** Immunoelectron microscopy of SWA11 internalisation by the SW2 cell line. **a**, Surface-bound antibody at the start of incubation. **b**, Gold particles within lysosomes after 2 h of incubation, untreated cells. **c**, Clumped gold particles present within lysosomes at 24 h, untreated cells. **d**, Antibody within vesicles of abnormal morphology in monensin-treated cells following 24 h of incubation. The bar line indicates a length of 0.1 μm.

### Discussion

The purpose of the present study was to examine in detail the cytotoxic action of the anti-SCLC IT SWA11-ricin A chain. The major findings were: (i) SWA11-ricin A chain consistently exerted potent cytotoxic effects against SCLC cell lines, (ii) the toxic action of the IT was selective for tumour cell lines expressing the target antigen, (iii) the most potent cytotoxic effects were against SCLC cell lines, (iv) cell intoxication by the IT was characterised by a lag phase followed by rapid kinetics of protein synthesis inhibition, (v) expression of the target antigen on a high proportion of SCLC cells ensured a high percentage elimination of clonogenic tumor cells, (vi) the activity of the IT was selectively enhanced by the carboxylic ionophore monensin but not by other potentiating agents, (vii) monensin accelerated the onset, and increased the rate, of protein synthesis inhibition, and (viii) monensin had no detectable influence on the rate of entry of the SWA11 Mab but may have transiently delayed transfer of the Mab to lysosomes.

The present study has conclusively demonstrated that the most important activity criteria demanded of a therapeutic IT are fulfilled by SWA11-ricin A chain. Firstly, SWA11-ricin A chain was highly active towards all the SCLC cell lines examined: against the three SCLC cell lines, which were of both classic and variant morphologies, the IT exerted similar potent cytotoxic effects. The comparable susceptibility of the SCLC cell lines reflects the high frequency of expression of the cluster w4 antigen both on SCLC tumours and cell lines (Souhami *et al.*, 1988) and suggests that a high

**Table III** Localisation of gold particles within lysosomes of the SW2 cell line treated with SWA11 in the presence or absence of monensin

| | Number of cell sections[a] | | | |
|---|---|---|---|---|
| *Time (h)* | *0*[b] | *1–5* | *6–10* | *>10* |
| 1–[c] | 4 | 6 | 0 | 0 |
| 1+ | 10 | 0 | 0 | 0 |
| 2– | 4 | 5 | 1 | 0 |
| 2+ | 5 | 4 | 0 | 1 |
| 4– | 4 | 4 | 0 | 2 |
| 4+ | 5 | 4 | 1 | 0 |
| 8– | 5 | 2 | 1 | 2 |
| 8+ | 3 | 2 | 4 | 1 |
| 24– | 1 | 3 | 1 | 4 |
| 24+ | 2 | 3 | 0 | 5 |

[a]Number of cell sections containing the given numbers of gold particles from ten cell sections examined in each case. [b]Number of lysosomal gold particles identified within a single cell section. [c]–/+ monensin at a concentration of $1 \times 10^{-7}$ M.

proportion of the target patient population with SCLC will have IT-sensitive disease.

Secondly, the cytotoxic action of the IT was selective for cluster w4 antigen-positive cell lines. The dependence of the anti-tumour activity of SWA11-ricin A chain upon binding to the cluster w4 antigen was demonstrated by three findings: (i) lack of any selective effect of an isotype-matched control IT of irrelevant specificity, (ii) inhibition of IT action by excess unconjugated SWA11 Mab, and (iii) lack of action of the IT on an antigen-negative tumour cell line at high concentration. The high activity of SWA11-ricin A chain against the SCLC cell lines in particular, which probably reflects the high level of expression of the target antigen on these lines compared with other tumour cell lines, suggests a potential therapeutic advantage in targeting SCLC.

Thirdly, the cytotoxic potency of the IT against SCLC is sufficient to allow potentially tumouricidal doses to be achieved in patients. Judging by the consistent $IC_{50}$ values determined for the SCLC cell lines, the potency of SWA11-ricin A chain exceeds that of ricin A chain ITs which have previously been used for clinical trials of IT therapy in other human solid tumours (Bjorn *et al.*, 1985; Embleton *et al.*, 1986; Spitler, 1987). The sensitivity of SCLC cell lines to the SWA11 IT in tissue culture is likely to be a good indication of the intrinsic susceptibility of SCLC tumours to the IT in patients because SCLC cell lines generally retain the biochemical and morphological characteristics of the tumours from which they are derived (Klein *et al.*, 1987; Vescio *et al.*, 1990; Tsai *et al.*, 1990).

Fourthly, the intoxication of SCLC cells by the IT occurred rapidly. The kinetics of protein synthesis inhibition were only some 3- to 4-fold slower than those of ricin toxin at equivalent concentration. Rapid intoxication of target cells is an important property of a therapeutic IT if escape of clonogenic tumour cells from the action of IT by cell division is to be minimised. The onset of effects upon protein synthesis was delayed by 4 h from the time of first exposure of the cells to IT. This lag presumably reflects the time required for the IT to bind to the cell surface, to be internalised and to become transported to the intracellular compartment involved with the translocation of the A chain to the cytosol.

Fifthly, SWA11-ricin A chain could selectively eliminate a high proportion of colony-forming SCLC cells. The expression of the target antigen on a high proportion of tumour cells is an important criterion for employment of ITs in cancer therapy because these agents rely on binding to target antigen-positive cells for their selective toxic action. The ability of the IT to eliminate in excess of 99.9% clonogenic tumour cells selectively suggests that a substantial effect on tumour growth could be achieved.

A number of agents such as lysosomotropic amines, carboxylic ionophores or calcium antagonists, have previously been shown to enhance the activity of ricin A chain ITs (Casellas & Jansen, 1988). Only the ionophore monensin was found to be capable of substantially potentiating the activity of SWA11-ricin A chain against the SW2 SCLC cell line. At a concentration of $1 \times 10^{-7}$ M, monensin decreased the $IC_{50}$ of the IT by 100-fold giving a potency of action comparable with that of ricin in the absence of ionophore. The effect of monensin was apparently exerted at an early stage following binding to the cell surface. Kinetic studies revealed that monensin not only enhanced the rate of protein synthesis inhibition but also eliminated the 4 h lag phase suggesting that either the rate or the route of IT internalisation had been rapidly affected. Studies with the SWA11 Mab could detect no influence of monensin on the rate of antibody internalisation. The only effect of ionophore detected on the route of internalisation was an apparently transient delay in the delivery of Mab to lysosomes consistent with the rapid effects seen on IT kinetics. In general, the findings of the present study correspond with those of previous studies which analysed the effects of monensin on different ITs and cellular targets (Casellas *et al.*, 1984; Carriere *et al.*, 1985; Manske *et al.*, 1986; Griffin *et al.*, 1987). It is likely that methods of enhancing IT activity using monensin *in vivo* (Hertler *et al.*, 1989; Colombatti *et al.*, 1990; Griffin & Raso, 1991) would also be applicable to SWA11-ricin A chain.

In conclusion, SWA11-ricin A chain is rapidly, potently and selectively toxic to a high proportion of SCLC tumour cells in tissue culture. These favourable properties suggest that the IT should be active against SCLC *in vivo*. Although high activity *in vitro* is no guarantee of good activity *in vivo*, it is nevertheless an obligatory requirement of an effective cytotoxic agent. The ability of monensin to selectively increase the potency of SWA11-ricin A chain after only a short duration of exposure suggests that potentiation of the IT *in vivo* might also be feasible. Experiments to determine the anti-tumour efficacy of an SWA11-ricin A chain IT designed for therapy have been undertaken in a nude mouse SCLC solid tumour xenograft model.

This study was supported by the Cancer Research Campaign, London, UK, and the Swiss Cancer League SOR302.89.2. E.J.D. wishes to thank the CRC for the award of a Research Studentship. We thank Miss C. Clarke for valued technical assistance and Dr P. Monaghan for his advice and critical comments on the manuscript.

### References

BECK, L.K., KANE, M.A. & BUNN, P.A. (1988). Innovative and future approaches to small cell lung cancer treatment. *Semin. Oncol.*, **15**, 300–314.

BJORN, M.J., RING, D. & FRANKEL, A. (1985). Evaluation of monoclonal antibodies for the development of breast cancer immunotoxins. *Cancer Res.*, **45**, 1214–1221.

CARNEY, D.N., GAZDAR, A.F., BEPLER, G., GUCCION, J.G., MARANGOS, P.J., MOODY, T.W., ZWEIG, M.H. & MINNA, J.D. (1985). Establishment and identification of small cell lung cancer cell lines having classic and variant features. *Cancer Res.*, **45**, 2913–2923.

CARRIERE, D., CASELLAS, P., RICHER, G., GROS, P. & JANSEN, F.K. (1985). Endocytosis of an antibody ricin A chain conjugate (immuno-A-toxin) adsorbed on colloidal gold. *Exp. Cell. Res.*, **156**, 327–340.

CASELLAS, P., BOURRIE, B.J.P., GROS, P. & JANSEN, F.K. (1984). Kinetics of cytotoxicity induced by immunotoxins: enhancement by lysosomotropic amines and carboxylic ionophores. *J. Biol. Chem.*, **259**, 9359–9364.

CASELLAS, P. & JANSEN, F.K. (1988). Immunotoxin enhancers. In *Immunotoxins*, Frankel, A.E., (ed.), p. 351–369. Kluwer: Boston.

COBB, P.W., LEMAISTRE, C.F. & JACKSON, L.A. (1991). Clinical evaluation of immunotoxins. *Cancer Bull.*, **43**, 233-239.

COLOMBATTI, M., DELL'ARCIPRETE, L., CHIGNOLA, R. & TRIDENTE, G. (1990). Carrier protein-monensin conjugates: enhancement of immunotoxin cytotoxicity and potential in tumor treatment. *Cancer Res.*, **50**, 1385-1391.

EMBLETON, M.J., BYERS, V.S., LEE, H.M., SCANNON, P., BLACKHALL, N.W. & BALDWIN, R.W. (1986). Sensitivity and selectivity of ricin toxin A chain-monoclonal antibody 791T/36 conjugates against human tumor cell lines. *Cancer Res.*, **46**, 5524-5528.

FRAKER, P.J. & SPECK, J.C. (1978). Protein and cell membrane iodination with a sparingly soluble chloroamide. *Biochem. Biophys. Res. Commun.*, **80**, 849-857.

GRIFFIN, T. & RASO, V. (1991). Monensin in lipid emulsion for the potentiation of ricin A chain immunotoxins. *Cancer Res.*, **51**, 4316-4322.

GRIFFIN, T.W., RICHARDSON, C., HOUSTON, L.L., LEPAGE, D., BOGDEN, A. & RASO, V. (1987). Antitumor activity of intraperitoneal immunotoxins in a nude mouse model of human malignant mesothelioma. *Cancer Res.*, **47**, 4266-4270.

HERTLER, A.A. & FRANKEL, A.E. (1991). Immunotoxins in the therapy of leukemias and lymphomas. *Cancer Invest.*, **9**, 211-219.

HERTLER, A.A., SCHLOSSMAN, D.M., BOROWITZ, M.J., BLYTHMAN, H.E., CASELLAS, P. & FRANKEL, A.E. (1989). An anti-CD5 immunotoxin for chronic lymphocytic leukemia: enhancement of cytotoxicity with human serum albumin-monensin. *Int. J. Cancer*, **43**, 215-219.

JOHNSON, E.A. & BROWN, B.W. Jr (1961). The Spearman estimator for serial dilution assays. *Biometrics*, **27**, 79-88.

KLEIN, J.C., ZURCHER, C. & VAN BEKKUM, D.W. (1987). Differential behaviour of human bronchial carcinoma cells in culture. *Cancer Res.*, **47**, 3251-3258.

MANSKE, J.M., BUCHSBAUM, D.J., AZEMOVE, S.M., HANNA, D.E. & VALLERA, D.A. (1986). Antigenic modulation by anti-CD5 immunotoxins. *J. Immunol.*, **136**, 4721-4728.

MINNA, J.D., PASS, H., GLATSTEIN, E.J. & IHDE, D. (1989). Cancer of the lung. In *Cancer: Principles and Practice of Oncology*, DeVita, V.T., Hellman, S.A. & Rosenberg, S.A. (eds.), p. 591-705. Lippincott: Philadelphia.

MONAGHAN, P., WHITEHEAD, R.H., PERUSINGHE, N. & O'HARE, M.J. (1985). An immunocytochemical and ultrastructural study of heterogeneity in the human breast carcinoma cell line PMC42. *Cancer Res.*, **45**, 5088-5097.

POSTMUS, P.E., DE LEIJ, L., VAN DER VEEN, A.Y., MESANDER, G., BUYS, C.H.C.M. & ELEMA, J.D. (1988). Two small cell lung cancer cell lines established from rigid bronchoscopic biopsies. *Eur. J. Canc. Clin. Oncol.*, **24**, 755-763.

SOUHAMI, R.L., BEVERLEY, P.C.L. & BOBROW, L. (1988) (eds.) Proceedings of the First International Workshop on Small Cell Lung Cancer Antigens. *Lung Cancer*, **4**, 1-116.

SPITLER, L.E. (1987). Phase I clinical trials with immunotoxins. In *Immunoconjugates: Antibody Conjugates in Radioimaging and Therapy of Cancer*. Vogel, C.W. (ed.), pp. 290-300. Oxford University Press: New York.

TSAI, C.M., IHDE, D.C., KADOYAMA, C., VENZON, D. & GAZDAR, A.F. (1990). Correlation of *in vitro* drug sensitivity testing of long-term small cell lung cancer cell lines with response and survival. *Eur. J. Cancer*, **26**, 1148-1152.

VESCIO, R.A., CONNORS, K.M., BORDIN, G.M., ROBB, J.A., YOUNGKIN, T., UMBREIT, J.N. & HOFFMAN, R.M. (1990). The distinction of small cell and non-small cell lung cancer by growth in native-state histoculture. *Cancer Res.*, **50**, 6095-6099.

WAWRZYNCZAK, E.J. (1991). Systemic immunotoxin therapy of cancer: advances and prospects. *Br. J. Cancer*, **64**, 624-630.

WAWRZYNCZAK, E.J., DERBYSHIRE, E.J., DE LEIJ, L., MENARD, S. & STAHEL, R.A. (1991*a*). Target-selective cytotoxic activity of immunotoxins to the common small cell lung cancer-associated antigens. *Lung Cancer*, **7**, Suppl., 182.

WAWRZYNCZAK, E.J., DERBYSHIRE, E.J., HENRY, R.V., PARNELL, G.D., SMITH, A., WAIBEL, R. & STAHEL, R.A. (1990*a*). Selective cytotoxic effects of a ricin A chain immunotoxin made with the monoclonal antibody SWA11 recognising a human small cell lung cancer antigen. *Br. J. Cancer*, **62**, 410-414.

WAWRZYNCZAK, E.J., DERBYSHIRE, E.J., HENRY, R.V., PARNELL, G.D., SMITH, A., WAIBEL, R. & STAHEL, R.A. (1991*b*). Cytotoxic activity of ricin A chain immunotoxins recognising cluster 1, w4 and 5A antigens associated with human small cell lung cancer. *Br. J. Cancer*, **63**, Suppl. XIV, 71-73.